# Okara: A Nutritionally Valuable By-product Able to Stabilize *Lactobacillus plantarum* during Freeze-drying, Spray-drying, and Storage

**DOI:** 10.3389/fmicb.2017.00641

**Published:** 2017-04-12

**Authors:** Gabriel Quintana, Esteban Gerbino, Andrea Gómez-Zavaglia

**Affiliations:** Center for Research and Development in Food Cryotechnology (CCT–CONICET La Plata)Buenos Aires, Argentina

**Keywords:** okara, *Lactobacillus plantarum*, fatty acid composition, preservation process, storage

## Abstract

Okara is a nutritionally valuable by-product produced in large quantities as result of soymilk elaboration. This work proposes its use as both culture and dehydration medium during freeze-drying, spray-drying, and storage of *Lactobacillus plantarum* CIDCA 83114. Whole and defatted okara were employed as culture media for *L. plantarum* CIDCA 83114. The growth kinetics were followed by plate counting and compared with those of bacteria grown in MRS broth (control). No significant differences in plate counting were observed in the three media. The fatty acid composition of bacteria grown in whole and defatted okara showed a noticeable increase in the unsaturated/saturated (U/S) fatty acid ratio, with regard to bacteria grown in MRS. This change was mainly due to the increase in polyunsaturated fatty acids, namely C18:2. For dehydration assays, cultures in the stationary phase were neutralized and freeze-dried (with or without the addition of 250 mM sucrose) or spray-dried. Bacteria were plate counted immediately after freeze-drying or spray-drying and during storage at 4°C for 90 days. Freeze-drying in whole okara conducted to the highest bacterial recovery. Regarding storage, spray-dried bacteria previously grown in whole and defatted okara showed higher plate counts than those grown in MRS. On the contrary, freeze-dried bacteria previously grown in all the three culture media were those with the lowest plate counts. The addition of sucrose to the dehydration media improved their recovery. The higher recovery of microorganisms grown in okara after freeze-drying and spray-drying processes and during storage was ascribed to both the presence of fiber and proteins in the dehydration media, and the increase in U/S fatty acids ratio in bacterial membranes. The obtained results support for the first time the use of okara as an innovative matrix to deliver *L. plantarum*. Considering that okara is an agro-waste obtained in large quantities, these results represent an innovative strategy to add it value, providing a symbiotic ingredient with promising industrial applications in the development of novel functional foods and feeds.

## Introduction

Okara is a white-yellowish puree remaining after filtration of the smashed soybeans seeds used for the production of soymilk (Stanojevic et al., 2013), and is produced in large quantities (about 1.1 kg per kilogram of soybean processed for soymilk production) (Li Y. et al., 2012). It is rich in high quality proteins, unsaturated lipids and dietary fiber, and also contains isoflavones, minerals and oligosaccharides (i.e., raffinose, stachyose) (Jimenez-Escrig et al., 2008; Song et al., 2009). Because of this nutritional richness, and the high water activity, okara is very prone to putrefaction. Although this agro-waste is generally used for animal feed, surplus is still burned as waste or dumped as landfill (Vong and Liu, 2016), which entails a significant disposal problem. For this reason, it is important to find novel strategies to add value to this material.

Several health benefits have been ascribed to okara, including antioxidant capacity, prevention of obesity and liver fat accumulation, decrease of cholesterol plasma levels and prevention of cardiovascular diseases (Jimenez-Escrig et al., 2008; Bedani et al., 2015; Vong and Liu, 2016). These properties make it a source of functional ingredients at a low cost (O’Toole, 1999; Li Y. et al., 2012). Okara was used as fermentation substrate to produce a variety of products for human consumption, including α-glucosidase inhibitor, β-fructofuranosidase, fibrinolytic enzymes, iturin A, edible fungi, chitosan, alcohol, biosurfactants, among others (Kitawaki et al., 2007; Li B. et al., 2012; Li Y. et al., 2012; Villanueva-Suárez et al., 2013; Zhu et al., 2014; Vong and Liu, 2016). It was also used to supplement culture media or probiotic-containing products (Bedani et al., 2013, 2014). Inoculation of okara with lactic acid bacteria has been reported as an efficient approach to inhibit the growth of spoilage microorganisms (O’Toole, 1999) and to add value to this by-product (Lê et al., 2004; Espinosa-Martos and Rupérez, 2009; Villanueva-Suárez et al., 2013; Vong and Liu, 2016). Considering this, okara could be used as a cost-effective culture medium for the production of large quantities of starters at an industrial level.

Among lactic acid bacteria, *Lactobacillus plantarum* is a flexible and versatile species that is encountered in a variety of environmental niches, including some dairy, meat, and many vegetable or plant fermentations (Kleerebezem et al., 2003). Several strains of *L. plantarum* have demonstrated probiotic and technological properties. For this reason, the strains of this species have been used for a large number of applications, namely the formulation of fortified bread and pasta (Capozzi et al., 2011), as malolactic fermentation starter cultures (Berbegal et al., 2016), for the fermentation of regional beverages (Yadav et al., 2016), as antibiotic replacement (Al-Madboly and Abdullah, 2015), and for the elimination of the undesirable biogenic amines in wines (Capozzi et al., 2012), among others. In particular, *L. plantarum* CIDCA 83114, a strain isolated from kefir grains, have demonstrated both probiotic and technological properties, including the inhibition of growth and/or activity of *Escherichia coli* O157:H7 and *Salmonella* (Golowczyc et al., 2011b; Kakisu et al., 2013), and the overcoming of thermal and dehydrating processes (Romano et al., 2014; Tavera-Quiroz et al., 2015). These properties make it a good candidate to grow in okara and add value to this by-product.

The production of lactic acid bacteria as industrial starters requires adequate stabilization processes to avoid bacterial damage or death. In general, starters are delivered to food companies, or directly to consumers, under the form of ready to use and highly concentrated products. The commercialization of these concentrated cultures requires the application of preservation (or stabilization) processes to increase their shelf-life. Among them, freeze-drying has been the most widely used process (Fonseca et al., 2015). Spray-drying is an alternative technique that conducts to the transformation of liquid systems into dry particulate powders when they come into direct contact with a drying medium (air) at high temperatures. It has been increasingly used to dehydrate lactic acid bacteria (Golowczyc et al., 2010, 2011a,b, 2013; Sosa et al., 2016). It is a cost-effective process, especially suitable for the production of large quantities of microorganisms (Golowczyc et al., 2010, 2011a,b, 2013; Sosa et al., 2016).

During the preservation processes, the decrease of water activity produces damages on the bacterial structures, decreasing their viability (Tymczyszyn et al., 2007a). To prevent these damages, sugars are generally used as protective agents (Carvalho et al., 2004), sucrose and trehalose being the most widely used among lactic acid bacteria. More recently more complex sugars, namely fructo and galacto-oligosaccharides, inuline, fiber, have been successfully used (Santos et al., 2014; Romano et al., 2015). Considering that okara includes about 50% of oligo and polysaccharides (Vong and Liu, 2016), its use as dehydration medium during preservation processes could provide protective compounds, avoiding the addition of external protectants.

Taking this background into account, the aim of this work was twofold: to use okara as culture medium for *L. plantarum* CIDCA 83114; and to evaluate the stabilizing capacity of okara during bacterial freeze-drying, spray-drying and storage at 4°C for 90 days.

## Materials and Methods

### Raw Material, Centesimal Composition, and Fourier Transform Infrared assays (FTIR)

Okara was obtained from Soyana S. H. (San Martín, Argentina). After being received, it was centrifuged five times to remove the excess of water. The sediment was frozen at -80°C for 48 h and then, freeze-dried on a Heto FD4 equipment (Heto Lab Equipment, Denmark) for 48 h (temperature of condenser: -50°C; chamber pressure: 0.04 mbar).

Moisture content of the freeze-dried okara was determined by measuring the weight loss upon drying in a vacuum oven at 70°C until constant weight (Association of Official Analytical Chemists [AOAC], 1980). Total nitrogen was determined using the Kjeldahl method (Association of Official Analytical Chemists [AOAC], 1995) (conversion factor to transform nitrogen into protein: 6.25). Lipids were assessed by extraction with diethyl ether solvent in a Soxhlet system (Association of Official Analytical Chemists [AOAC], 1995). Ash content was determined by carbonization of the dried samples followed by incineration in a muffle furnace at 550°C. Dietary fiber was assessed using Megazyme testkit K-ACHDF 11/08 (Megazyme International Ireland Limited, Bray, Ireland) according to the manufacturer’s procedure (McCleary, 2007). The centesimal composition was expressed in grams per 100 g of dry basis. The centesimal composition of defatted okara was determined after lipids’ extraction (Association of Official Analytical Chemists [AOAC], 1995).

Fourier transform infrared spectroscopy spectra of freeze-dried whole and defatted okara were registered in the 4000–500 cm^-1^ range on KBr pellets, prepared in a 1:200 okara-KBr ratio. They were recorded in a transmission mode by co-adding 64 scans with 4 cm^-1^ spectral resolution, on a Thermo Nicolet iS10 spectrometer (Thermo Scientific, Waltham, MA, USA).

Whole and defatted okara were suspended in distilled water to obtain 5% w/v suspensions. The suspensions were homogenized in an Ultra Turrax T25 (IKA, Staufen im Breisgau, Germany) and autoclaved for 15 min at 121°C. They were then used as culture media.

### Bacterial Strain, Culture Conditions, and Growth Kinetics

*Lactobacillus plantarum* CIDCA 83114 was isolated from kefir grains (Garrote et al., 2001) maintained frozen at -80°C in 120 g/L non-fat milk solids (Difco, USA). Microbial cells were activated for 24 h in MRS broth (de Man et al., 1960) at 37°C in aerobic conditions. The resulting culture was inoculated (1% v/v) in fresh MRS broth and incubated in the same conditions. Cultures in the stationary phase [18 h incubation and ∼1 × 10^13^ colony forming units per mL (CFU/mL)] were used to inoculate 100 mL whole and defatted okara (inoculum concentration 2% v/v). They were then incubated at 37°C with shaking. In a parallel assay, microorganisms were grown in MRS broth in the same conditions (control). The three growth kinetics were followed by plate counting in MRS agar every 1 h, and results were expressed in CFU/mL.

### Extraction of Lipids from Bacteria

Microorganisms were grown in okara and defatted okara at 37°C. Once attained the stationary phase, cultures were treated with 2% w/v EDTA pH 12 for 10 min to sequester calcium from okara and partly solubilize insoluble structures (Holt, 1982). After the treatment with EDTA, cultures were centrifuged, supernatants were discarded and bacterial pellets were used for lipids’ extraction. Controls: bacteria in the stationary phase grown in MRS, harvested by centrifugation, and washed with PBS [(K_2_HPO_4_ 0.144 g/L; NaCl 9.00 g/L; Na_2_HPO_4_ g/L), pH 7].

Lipids were extracted according to the modified Bligh and Dyer method (Marinetti, 1993). Briefly, bacterial pellets from microorganisms grown in whole and defatted okara and in MRS were suspended in chloroform-methanol-water (1:2:0.8 v/v/v) (4.75 mL per g of cells) for 12 h at 4°C, and then centrifuged at 8000 *g* for 10 min at 10°C. The supernatants were collected, and a second extraction on bacterial pellets was carried out. Both supernatants were mixed and chloroform-water (1:1) was added (12.5 mL per g of bacteria). The final mixture was centrifuged at 8000 *g* for 20 min. The chloroform phase was collected and dried under vacuum (Rotavapor^®^ RE 120 – Büchi, Flawil, Switzerland). Lipids were dissolved in chloroform (final concentration 3 mg/mL) and stored at -20°C for up to 2 weeks.

### Characterization of Fatty Acids

The FAME were prepared by adding 2 mL sulfuric acid (20 g/L in methanol) to 3 mg bacterial lipids, heating at 60°C for 2 h, extracting the esters with 1 mL chloroform-water (2:0.7 v/v) and washing twice with 0.7 mL water. The obtained FAME were analyzed on a gas chromatograph interfaced with a mass spectrometer detector (Shimadzu QP 5050A, Tokyo, Japan) using capillary column ZB-5 (30 m × 0.25 mm). The analysis conditions were: injection temperature 250°C, detector temperature 280°C, and column temperature initially 100°C increased to 280°C at 6°C min^-1^. The FAME were identified by mass spectrometry (GC–MS). The fatty acid composition was determined by considering the relative contribution of each peak area with regard to the sum of the areas corresponding to all peaks. Results were expressed as % of the total area.

### Freeze-drying

Microorganisms were grown in okara and defatted okara at 37°C. Aliquots of 1 mL containing *L. plantarum* CIDCA 83114 were collected in the stationary phase and neutralized with NaOH to pH 7. Then, they were transferred into 5 mL glass vials under aseptic conditions and frozen at -80°C for 48 h. The freeze-drying process was carried out at -50°C using a Heto FD4 freeze drier (Heto Lab Equipment, Denmark) and lasted 48 h. In parallel assays, sucrose was added to the neutralized okara (whole and defatted) containing *L. plantarum* CIDCA 83114 (final concentration of sucrose: 250 mM). Freeze-drying conditions were the same as those described above. In turn, microorganisms grown in MRS broth were harvested, neutralized and resuspended in 250 mM sucrose or in PBS. Freeze-drying process was carried out as described for bacteria grown in okara. Results were expressed as log N/N_0_, where N and N_0_ were the CFU/mL after and before freeze-drying, respectively.

### Spray-drying

In a parallel experiment, 100 mL of okara suspensions (whole and defatted) containing *L. plantarum* CIDCA 83114 in the stationary phase were neutralized and spray-dried in a laboratory-scale spray-dryer (Büchi B290 mini spray-dryer, Flawil, Switzerland) at a constant air inlet temperature of 180°C and an outlet temperature of 65–70°C. Microorganisms grown in MRS broth were harvested neutralized and resuspended in PBS prior spray-drying. Results were expressed as log N/N_0_, where N and N_0_ were the CFU/mL after and before spray-drying, respectively.

### Storage Conditions

After freeze-drying or spray-drying, samples were stored in silica gel desiccators at 4°C for 90 days. During storage, and at regular intervals, freeze-dried and spray-dried samples were rehydrated in PBS, homogenized for 1 min in a vortex mixer and maintained at room temperature for 30 min. Bacterial suspensions were serially diluted and plate counted on MRS agar, after incubation at 37°C for 48 h in aerobic conditions.

### Mathematical Modeling

Storage plots were fitted using lineal and non-lineal mathematical regressions according to Eqs 1 and 2, respectively:

$$\text{Log N/}{\text{N}}_{\text{0}}=\text{ -kt}$$

where N is the CFU/mL at a given time of storage, N_0_ is the CFU/mL at time equal to 0 (immediately after the preservation process), t is the time of storage (expressed in days) and k is the constant of bacterial inactivation (expressed in days^-1^).

Equation 2 describes non-lineal decays as:

$$\text{Log N/}{\text{N}}_{\text{0}}=\text{-A }{\text{e}}^{\text{-k-t}}+\text{A}$$

where N is the CFU/mL at a given time of storage, N_0_ is the CFU/mL at time equal to 0 (immediately after the process), t is the time of storage (expressed in days), k is the constant of bacterial inactivation (expressed in days^-1^) and A is the value of Log N/N_0_ at the *plateau*.

### Water Activity Measurements

The a_w_ of freeze-dried and spray-dried samples was determined using an Aqualab water activity instrument (Aqualab, Model Series 3TE, USA). The equipment was calibrated using standard salt solutions provided by the manufacturer.

### Statistical Analysis and Reproducibility

All experiments were carried out on duplicate samples using three independent cultures of bacteria. The relative differences were reproducible independently on the cultures used. Analysis of variance (ANOVA) of cultivable bacteria corresponding to the different treatments was performed using the statistical program GraphPad Prism version 5.01 for Windows (GraphPad Software, Inc., San Diego, CA, USA 2007). Comparison of means by Tukey methods were tested, and if *p* < 0.05 the difference was considered statistically significant.

## Results and Discussion

**FIGURE 1:**
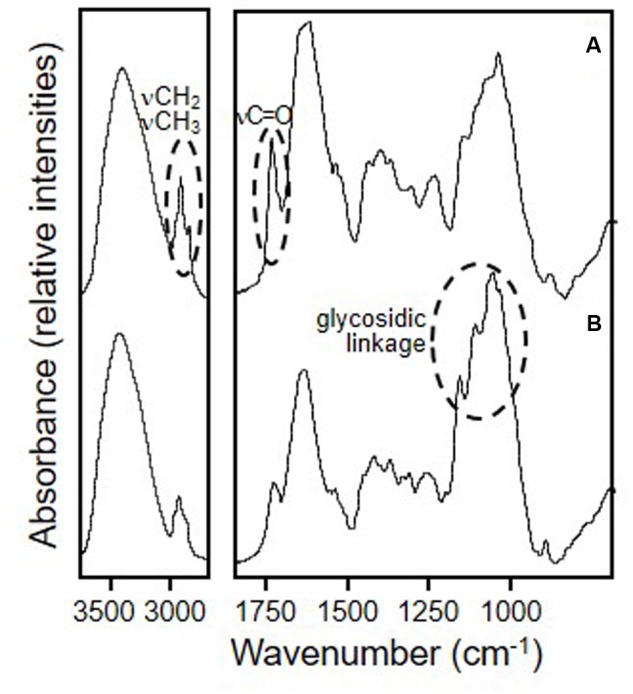
**Fourier transform infrared spectroscopy spectra of freeze-dried okara.** **(A)** whole okara; **(B)** defatted okara. Slashed circles denote the main differences between both spectra.

The centesimal composition of whole and defatted okara is presented in **Table 1**, and was comparable to that reported for okara in other works (Vong and Liu, 2016). The FTIR spectra corresponding to whole and defatted okara are shown in **Figure 1**. The main differences observed in the spectra are related with the lipid and carbohydrate bands. In fact, a clear decrease of the typical narrow νCH_2_ bands corresponding to hydrocarbon lipid chains (2931 and 2860 cm^-1^) and the νC = O band at 1750 cm^-1^ ascribed to the carbonyl groups of fatty acids, was observed in defatted okara. In addition, a noticeable increase of the relative intensity of the bands in the 1200–900 cm^-1^ region, ascribed to the absorption of the C-O-C glycosidic linkage, was observed (Romano et al., 2014) (**Figure 1**). This reflects the higher contribution of fiber in defatted okara, which is consistent with the information shown in **Table 1**.

**Table 1:** Centesimal composition of freeze-dried whole and defatted okara.

Centesimal composition	Whole okara (g/100 g d.b.)	Defatted okara (g/100 g d.b.)
Protein	20.90 ± 1.88	23.25 ± 2.09
Lipids	13.40 ± 1.24 (a)	3.67 ± 0.34 (b)
Whole fiber	54.51 ± 2.43	60.64 ± 2.70
Ash	1.54 ± 0.67	1.71 ± 0.75
Humidity	9.64 ± 2.30	10.73 ± 2.69


*Different letters (a, b) denote statistically significant differences (*p* < 0.05).*

**FIGURE 2:**
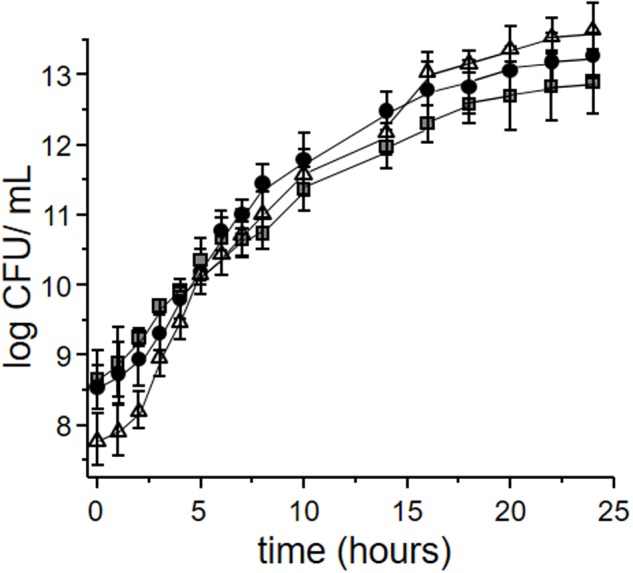
**Growth kinetics of *Lactobacillus plantarum* CIDCA 83114 in whole okara (black circles); defatted okara (gray squares); MRS (control) (white triangles)**.

**Figure 2** depicts the growth kinetics of *L. plantarum* CIDCA 83114 in okara, defatted okara and MRS. No significant differences were observed for microorganisms grown in whole and defatted okara with regard to the control grown in MRS (*p* > 0.05), demonstrating that both whole and defatted okara are as adequate as MRS to grow *L. plantarum* CIDCA 83114. Because of the large availability of okara, using okara as culture medium represents a clear advantage for the large scale production of starters, as costs can be substantially reduced. It has been reported that certain strains of lactobacilli and bifidobacteria ferment okara, and for this reason, okara has been proposed as a potential source of prebiotic fiber (Espinosa-Martos and Rupérez, 2009).

The composition of culture media strongly determines the composition of bacterial lipid membranes (Smittle et al., 1974; Tymczyszyn et al., 2005), and this latter is in turn related with their stability during preservation processes (Gomez-Zavaglia et al., 2000). For this reason, efforts were made to determine the fatty acid composition of okara lipids, which included C18:2 (54.61%), C18:1 (21.34%), C16:0 (11.57%), and C18:3 (7.97%) fatty acids, in agreement with the information published by Peñalvo et al. (2004). Taking into account the high contribution of C18:2 and C18:1, okara can be considered as a culture medium enriched in unsaturated fatty acids. Because of the high nutritional value and the technological properties, we have foreseen that okara oil can be used for different applications, thus remaining defatted okara as a final by-product. For this reason, and considering that defatted okara may be a real by-product in case okara oil is used as food ingredient, we decided to include it in this work.

**Table 2** shows the fatty acid composition of *L. plantarum* CIDCA 83114 grown whole and defatted okara and in MRS (control). *L. plantarum* CIDCA 83114 grown in MRS had mainly C16:0, C18:1 and cycC19:0, with an U/S ratio of 0.63. Growing microorganisms in okara induced noticeable changes in the fatty acid composition, mainly due to the increase in the polyunsaturated fatty acids (i.e., C18:2) (**Table 2**). The contribution of other fatty acids, like C16:1 and cycC19:0, strongly dropped for bacteria grown in okara. A moderate decrease of C16:0 and C18:1 was also observed. As result of these changes, an inversion in the U/S ratio was observed for microorganisms grown in whole and defatted okara. Moreover, due to the high contribution of C18:2, the PUFA/MUFA ratio also increased with regard to the controls grown in MRS (**Table 2**). It has been reported that growing bacteria in media enriched with unsaturated fatty acids induces an increase in the U/S fatty acids ratio, which prevents lipids from being closely packed, and thus increases membrane fluidity (Muller et al., 2011; Hansen et al., 2015). Hansen et al. (2015) reported that the addition of 10 mg/L C18:2 to the growth medium promotes its incorporation into bacterial membranes. If considered that: (i) defatted okara had a lipid concentration of 3.67 ± 0.34 g/100 g d.b. (**Table 1**), (ii) bacteria were grown in 5% w/v okara suspensions (see Materials and Methods) and (iii) okara fatty acids included 54.61% C18:2, it can be calculated that microorganisms grown in defatted okara were cultured in the presence of 1 mg/mL C18:2. This concentration was higher than that reported by Hansen et al. (2015), thus explaining the increase of PUFA/MUFA ratio in microorganisms grown in defatted okara (**Table 2**). The same rationalization can be carried out for microorganisms grown in whole okara, which explains the changes observed in the fatty acid composition (**Table 2**).

**Table 2:** Fatty acid composition of *Lactobacillus plantarum* CIDCA 83114 grown in MRS, whole and defatted okara.

Ratio	MRS	Whole okara	Defatted okara
			
C14:0	4.68 ± 0.23 (a1)^a^	2.01 ± 0.10 (a1)	1.00 ± 0.05 (a1)
C16:0	31.75 ± 1.59 (a2)	25.83 ± 1.29 (b2)	21.10 ± 1.06 (b2)
C16:1	6.91 ± 0.35 (a3)	n.d.^b^	0.63 ± 0.03 (b3)
C18:0	4.83 ± 0.24 (a4)	11.08 ± 0.56 (b4)	6.93 ± 0.35 (a4)
C18:1	30.09 ± 1.52 (a5)	16.94 ± 0.85 (b5)	20.00 ± 1.00 (b5)
C18:2	0.67 ± 0.03 (a6)	44.14 ± 2.21 (b6)	47.32 ± 2.37 (c6)
cycC19:0	18.78 ± 0.94 (a7)	n.d.	0.49 ± 0.02 (b7)
U/S^c^	0.63	1.57	2.30
PUFA/MUFA^d^	0.02	2.61	2.29


*^a^Analysis of variance has been carried out on rows. They were indicated as: a1, a2, a3, b1, b2, b3, etc. ^b^n.d, not detected. ^c^U/S: unsaturated/saturated ratio. Unsaturated fatty acids include C16:1, C18:1, and C18:2. Saturated fatty acids include C14:0, C16:0, C18:0, and cycC19:0. ^d^PUFA/MUFA: polyunsaturated/monounsaturated ratio. Polyunsaturated fatty acids include C18:2 and monounsaturated fatty acids include C16:1 and C18:1.*

**FIGURE 3:**
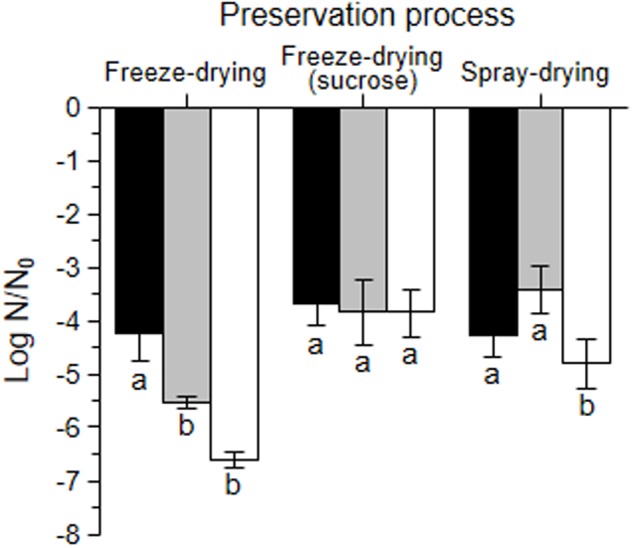
**Log N/N_0_ of *L. plantarum* CIDCA 83114 grown in whole okara (black bars), defatted okara (gray bars) and in MRS (white bars) after freeze-drying (left group of bars), freeze-drying with sucrose (middle group of bars) and spray-draying (right group of bars).** N_0_ corresponds to viable plate counts of fresh cultures in the stationary phase before each treatment, and was 13.60 ± 0.30 log CFU/mL for whole okara, 13.35 ± 0.53 log CFU/mL for defatted okara, and 13.68 ± 0.42 log CFU/mL for MRS. N corresponds to viable plate counts immediately after each treatment (log CFU/mL). Different letters (a, b) denote statistically significant differences (*p* < 0.05).

The effect of the growth and dehydration conditions on bacterial recovery was further analyzed. Freeze-dried microorganisms (with and without sucrose) had lower a_w_ than spray-dried ones, and bacteria grown in okara (whole and defatted), lower values than bacteria grown in MRS (**Table 3**). Bacteria grown in whole okara were those showing the lowest loss of viability after freeze-drying (left groups of bars in **Figure 3**). The log N/N_0_ value for freeze-dried bacteria grown in defatted okara was significantly lower (*p* < 0.05), and comparable with that of bacteria grown in MRS and suspended in PBS (*p* > 0.05). The addition of sucrose in the dehydration media improved the recovery of microorganisms grown in defatted okara and MRS after freeze-drying (*p* < 0.05) and did not have a significant effect on bacteria grown in whole okara (*p* > 0.05) (middle group of bars). It is interesting to note that the growth medium did not induce significant differences after freeze-drying with sucrose (*p* > 0.05) (middle group of bars). For the spray-drying process, the loss of viability for microorganisms grown in whole and defatted okara was significantly lower than that of bacteria grown in MRS and suspended in PBS (*p* < 0.05) (right group of bars).

**Table 3:** Water activity and inactivation constants of *L. plantarum* CIDCA 83114 grown in okara (whole and defatted) and MRS, and then freeze-dried (with and without 250 mM sucrose) or spray-dried.

Growth medium	Dehydration condition	a_w_^a^	Fitting		
			
			No significant decay	Linear^b^	Non-linear^c^
Whole okara	Freeze-drying	0.2544		k: 0.053 days^-1^ (*R*^2^: 0.9618)	
	Freeze-drying with sucrose	0.2466	X^d^		
	Spray-drying	0.3754	X		

Defatted okara	Freeze-drying	0.2635			k: 0.04254 days^-1^ A: 2.994 (*R*^2^: 0.9239)
	Freeze-drying with sucrose	0.2525	X		
	Spray-drying	0.3904			k: 0.03561 days^-1^ A: 2.881 (*R*^2^: 0.9536)

MRS	Freeze-drying	0.4141		k: 0.0780 days^-1^ (*R*^2^: 0.9419)	
	Freeze-drying with sucrose	0.2884		k: 0.035 days^-1^ (*R*^2^: 0.9622)	
	Spray-drying	0.4772			k: 0.1216 days^-1^ A: 4.082 (*R*^2^: 0.9193)


*^a^Water activity after the treatment. ^b^Linear fitting is described by Eq. 1: Log N/N_*0*_ = -*k*t. *N* = CFU/mL, where *N_*0*_* = CFU/mL at time equal to, *t* = time of storage in days; *k* = constant of bacterial inactivation, expressed in days^-*1*^ (see text). ^c^Non-linear fitting is described by Eq. 2: y = -A e^-*k-t*^+A, where *N* = CFU/mL at a given time of storage; *N_*0*_* = CFU/mL at time equal to 0; *t* = time of storage expressed in days, *k* = constant of bacterial inactivation expressed in days^-*1*^; A = value of Log N/N_*0*_ once attained the *plateau*. ^d^X denote no significant decay during storage.*

The effect of the growth medium on the preservation processes was also analyzed. Microorganisms grown in whole okara showed no significant differences in the Log N/N_0_ after the three treatments (*p* > 0.05) (**Figure 3**). In turn, for bacteria grown in defatted okara and in MRS, spray-drying and freeze-drying with sucrose were significantly less harmful processes than freeze-drying without sucrose (*p* < 0.05).

**FIGURE 4:**
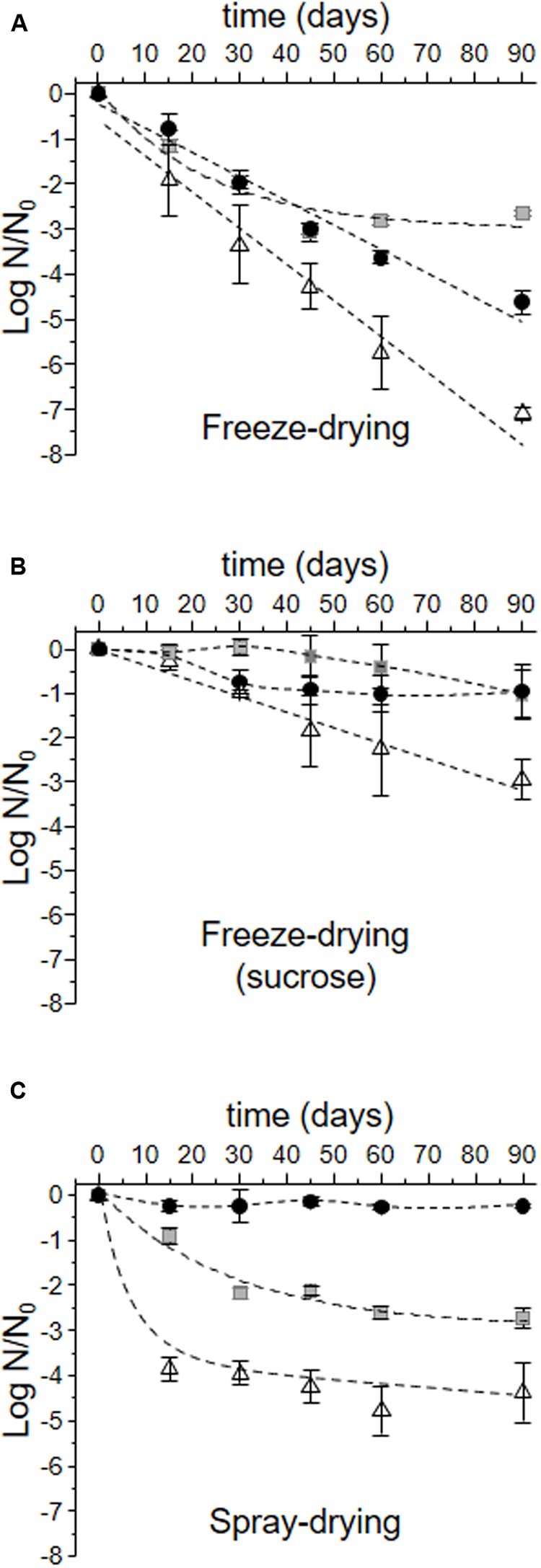
**Log N/N_0_ of *L. plantarum* CIDCA 83114 during storage.** N_0_: viability obtained immediately after each preservation process (in log CFU/mL) and correspond to the values of N used in **Figure 3**. N: viability after each time of storage (log CFU/mL). Full black circles: bacteria grown in whole okara; gray squares: bacteria grown in defatted okara; white triangles: bacteria grown in MRS. **(A)** Freeze-drying; **(B)** Freeze-drying in the presence of sucrose; **(C)** Spray-drying. Dash lines show the mathematical regressions. In the cases of no significant decays (see **Table 3**), experimental values were just connected.

The viability loss of *L. plantarum* CIDCA 83114 during storage at 4°C is shown in **Figure 4**. Freeze-drying was the most harmful process for *L. plantarum* CIDCA 83114 grown in the three culture media (**Figure 4A**). After 90 days of storage at 4°C, the viability of microorganisms grown in whole and defatted okara decayed 4.62 ± 0.25 and 2.65 ± 0.06 logarithmic units, respectively with regard to the corresponding viability obtained immediately after freeze-drying, considered as N_0_ (time equal to 0). A significantly more deleterious effect was observed for microorganisms grown in MRS (<0.05), as a decrease of 7.10 ± 0.14 logarithmic units was observed after 90 days of storage at 4°C. The addition of sucrose to the dehydration media had a protective effect during storage in all the three conditions assayed (**Figure 4B**). In this condition, microorganisms grown in whole and defatted okara did not show a significant decrease of viability with regard of the corresponding values obtained immediately after freeze-drying (*p* > 0.05) (**Figure 4B**). Although viability of microorganisms grown in MRS significantly decreased during storage (*p* < 0.05), this decrease was lower than that observed for bacteria grown in MRS and freeze-dried without sucrose (**Figure 4A**). In turn, spray-drying resulted an adequate process to stabilize bacteria grown in whole and defatted okara for 90 days at 4°C (**Figure 4C**). In this condition, no significant viability loss was observed for bacteria grown in whole okara (*p* < 0.05), and a loss of 2.71 ± 0.22 logarithmic units, for bacteria grown in defatted okara. Growing bacteria in MRS before spray-drying induced the highest drop of viability (4.15 ± 0.66 logarithmic units after 90 days of storage).

Polyhydroxy compounds, namely sugars and fiber, are generally used as protective compounds during freeze-drying, spray-drying and storage of lactic acid bacteria (Tymczyszyn et al., 2008; Santos et al., 2014; Romano et al., 2015). Fiber and oligosaccharides present in okara could improve the bacterial capacity to overcome freeze-drying and spray-drying processes and also stabilize microorganisms during storage (**Figures 3**, **4**) (Hincha et al., 2003; Sosa et al., 2016). The addition of sucrose in the dehydration medium improved the recovery of microorganisms grown in defatted okara and in MRS (**Figure 3**), which was an expected result considering the reported protective effect of sucrose during preservation of lactic acid bacteria (Tymczyszyn et al., 2007a,b). In this regard, it is worth to mention that although there are recent articles reporting the sucrose metabolism in *L. plantarum* strains (Chen et al., 2014; Mendoza-Llerenas et al., 2016), in this work, *L. plantarum* CIDCA 83114 is exposed to sucrose in non-growing conditions. Thus, sucrose is not hydrolyzed and acts as a protective agent.

The mathematical fitting of the storage graphs allowed the description of three different groups:

*(a) no significant viability decays after 90 days of storage:* this group included bacteria grown in whole okara and freeze-dried with sucrose or spray-dried, and microorganisms grown in defatted okara and freeze-dried with sucrose (**Figure 4** and **Table 3**).
*(b) linear decay after 90 days of storage:* this group included bacteria grown in okara and freeze-dried without sucrose, and those grown in MRS, freeze-dried with or without sucrose (**Table 3**). Eq. 1 describes the behavior of this group.
*(c) non-linear decay after 90 days of storage:* this group is composed of bacteria grown in defatted okara freeze-dried without sucrose or spray-dried, and bacteria grown in MRS and then, spray-dried (**Table 3**). Eq. 2 describes the behavior of this group.

For probiotic products, the European Food Safety Agency (EFSA) requires a bacterial viability of at least 10^6^–10^7^ CFU/g at the moment of being consumed (Hill et al., 2014). The *k* parameters obtained from Eqs 1 and 2 (**Table 3**) represent important tools to determine these values and in consequence, the shelf-life of *L. plantarum* CIDCA 83114 stabilized in okara matrices at the storage conditions.

## Conclusion

Fermenting okara with *L. plantarum* CIDCA 83114 appears as an interesting strategy to deal with okara spoilage and disposal. In addition, dehydrating microorganisms in okara resulted in an adequate approach to stabilize *L. plantarum* CIDCA 83114. In particular, the successful stabilization of fermented okara by spray-drying appears as a cost-effective solution for the production of starters at an industrial level with a low-energy consumption. The composition of okara had a strong influence on bacterial recovery after freeze-drying, spray-drying and storage: the presence of PUFAs induced an increase in the U/S and PUFA/MUFA ratios of lipid membranes, increasing membrane fluidity, and the fiber and oligosaccharides present in okara acted as protectants during dehydration and storage.

Using a by-product produced in large quantities as culture medium provides a cost-effective alternative for the production of starters at large scale. In addition, it provides a valuable solution for industrials, as they can solve two problems: okara disposal and availability of low-cost culture media. Regarding a nutritional perspective, okara provides high quality nutrients as vehicles of potentially probiotic microorganisms, whose importance in human and animal health is out of question. Taking this into account, the results support the use of okara matrices as suitable vehicles for probiotic strains, with promising applications in the development of novel functional foods and feeds.

## Author Contributions

GQ and EG did the experimental work. EG and AG-Z conceived the work, analyzed and discussed results and wrote the manuscript.

## Conflict of Interest Statement

The authors declare that the research was conducted in the absence of any commercial or financial relationships that could be construed as a potential conflict of interest.

## Abbreviations

a_w_: water activity
CFUs: cell forming units
cyc19: 0 cyclopropane fatty acid (methyleneoctadecanoic acid)
d.b.: dry basis
EDTA: ethylenediaminetetraacetic acid
FAMEs: fatty acids methyl esters
FTIR: Fourier transform infrared spectroscopy
MUFAs: monounsaturated fatty acids
PBS: phosphate saline buffer
PUFAs: polyunsaturated fatty acids
U/S: unsaturated/saturated
